# Bone regeneration capacity of newly developed spherical magnesium phosphate cement granules

**DOI:** 10.1007/s00784-021-04231-w

**Published:** 2021-10-23

**Authors:** Andreas Fuchs, Dorothea Kreczy, Theresa Brückner, Uwe Gbureck, Philipp Stahlhut, Melanie Bengel, Andreas Hoess, Berthold Nies, Julia Bator, Uwe Klammert, Christian Linz, Andrea Ewald

**Affiliations:** 1 Department of Oral & Maxillofacial Plastic Surgery, University Hospital Würzburg, Pleicherwall 2, 97070 Würzburg, Germany; 2 Department for Functional Materials in Medicine and Dentistry, University Hospital Würzburg, Pleicherwall 2, 97070 Würzburg, Germany; 3 INNOTERE GmbH, Meissner Strasse 191, 01445 Radebeul, Germany

**Keywords:** Calcium-magnesium phosphate cement, Cement pastes, Prefabricated granules, Bone replacement material, Implantation

## Abstract

**Objectives:**

Magnesium phosphate–based cements begin to catch more attention as bone substitute materials and especially as alternatives for the more commonly used calcium phosphates. In bone substitutes for augmentation purposes, atraumatic materials with good biocompatibility and resorbability are favorable. In the current study, we describe the in vivo testing of novel bone augmentation materials in form of spherical granules based on a calcium-doped magnesium phosphate (CaMgP) cement.

**Materials and methods:**

Granules with diameters between 500 and 710 μm were fabricated via the emulsification of CaMgP cement pastes in a lipophilic liquid. As basic material, two different CaMgP formulations were used. The obtained granules were implanted into drill hole defects at the distal femoral condyle of 27 New Zealand white rabbits for 6 and 12 weeks. After explantation, the femora were examined via X-ray diffraction analysis, histological staining, radiological examination, and EDX measurement.

**Results:**

Both granule types display excellent biocompatibility without any signs of inflammation and allow for proper bone healing without the interposition of connective tissue. CaMgP granules show a fast and continuous degradation and enable fully adequate bone regeneration.

**Conclusions:**

Due to their biocompatibility, their degradation behavior, and their completely spherical morphology, these CaMgP granules present a promising bone substitute material for bone augmentation procedures, especially in sensitive areas.

**Clinical relevance:**

The mostly insufficient local bone supply after tooth extractions complicates prosthetic dental restoration or makes it even impossible. Therefore, bone augmentation procedures are oftentimes inevitable. Spherical CaMgP granules may represent a valuable bone replacement material in many situations.

## Introduction

Despite a wide range of available bone replacement materials, the ideal bone substitute is yet to be found. Especially after tooth extractions, bone regeneration is regularly decreased due to an atrophy of the alveolar ridge of the maxilla or mandible. This complicates the insertion of dental implants or makes it even impossible [1]. A well-established procedure to overcome this problem is guided bone regeneration (GBR). In this, a combination of bone substitute materials and membranes is used in order to prevent the ingrowth of fast proliferating connective tissue into the bone defect and to allow for proper bone formation [2]. Generally, autologous, allogenic, xenogeneic, or synthetic (ceramics and polymers) bone replacement materials are currently available as bone grafts for craniomaxillofacial bone regeneration [3]. Nowadays, a number of different synthetic materials have been introduced as bone substitutes, for example, calcium phosphate ceramics [4], bioglasses [5], or resorbable polymers on a polylactide basis [6]. Such alloplastic materials are characterized by a constant quality regarding their structure and composition, which makes the biological reaction on their implantation site assessable. Due to their chemical similarity to the mineral phase of bone, calcium phosphates are currently mostly preferred as bone substitutes [7]. In this, established application forms comprise solid ceramic implants, coatings on metal implants, hydraulic cement, and particulate granules [8, 9]. Especially the latter are widely used in dental applications as bone fillers and are commercially available as medical products mainly based on hydroxyapatite, beta-tri-calcium phosphates, or biphasic mixtures of both. However, the individual particles of such granulate bone substitutes often exhibit sharp edges as they are made from bulk material by crushing and sieving. In some indications, such as the sinus floor elevation, this can increase the risk of a perforation of the mucosa of the maxillary sinus, which may consecutively lead to a loss of the augmentation material due to infection or displacement.

An important aspect of the bone regeneration capacity of alloplastic bone substitutes is their resorption potential in vivo. This is determined by their thermodynamic solubility (passive resorption) and the activity of adherent osteoclastic cells (active resorption) [10]. In this respect, magnesium phosphates became interesting as bone replacement material alternatives because minerals such as struvite (MgNH_4_PO_4_ × 6H_2_O) or newberyite (MgHPO_4_ × 3H_2_O) display a better solubility compared to conventional calcium phosphates as well as a good biocompatibility [11, 12]. Released magnesium ions are hereby assumed to stimulate bone remodeling and therefore lead to a faster bone ingrowth into the defect and resorption of the replacement material [13]. Magnesium phosphate–based bone substitutes are mostly processed via a cementitious reaction in which mineral powders are mixed with an aqueous solution. For example, magnesium phosphate–based starting powders (e.g., Mg_3_(PO_4_)_2_) can react with ammonium ions ((NH_4_)_2_HPO_4_) at a neutral pH value to form the mineral struvite [14]. With a solubility product of 2.12 × 10^−13^ in a pH range of 7.01–9.62, struvite exhibits a much higher resorbability than currently used hydroxyapatite or tricalcium phosphate–based granulate materials making it an ideal bone replacement material.

The aim of this study was to evaluate spherical granules made of magnesium phosphates with calcium substitution of the formula Ca_x_Mg_(3-x)_(PO_4_)_2_ with x = 0.25 and 0.75 respectively for clinical applications. Based on improved biological effects as shown before [15], a partial substitution of the magnesium phosphate raw powder by calcium ions was performed and two different types of raw powders were used. These compositions were expected to resorb much better than calcium phosphate granules and simultaneously enhance new bone formation. The obtained granules with different Mg:Ca ratios were implanted into femoral defects of New Zealand white rabbits. Bone regeneration capacity and resorbability of the granules were evaluated by element and structure analysis as well as radiological and histological examination after 6 and 12 weeks post-implantation.

## Materials and methods

### Cement granules preparation

The production of the Ca_x_Mg_(3-x)_(PO_4_)_2_ raw powder for the fabrication of granules was performed as described previously by our working group [15, 16]. Powders with the composition of Ca_0.25_Mg_2.75_(PO_4_)_2_ and Ca_0.75_Mg_2.25_(PO_4_)_2_ were synthesized as cement raw materials by sintering mixtures of MgHPO_4_ × 3H_2_O (Sigma-Aldrich, München, Germany), CaHPO_4_ (Baker, Schwerte, Germany), CaCO_3_ (Merck, Darmstadt, Germany), and Mg(OH)_2_ (VWR, Darmstadt, Germany) in appropriate stoichiometric ratios at 1100 °C for 5 h, respectively. The sintered cakes were manually crushed, sieved < 125 μm, and ground dry for 1 h in a ball mill (Retsch PM400, Retsch GmbH, Idar-Oberstein, Germany). Granules were produced via an emulsion process of CaMgP cement pastes in an oil phase. This oil phase consisted of Tween 80 (3.0 wt.% of the cement powder) that was mixed with viscous paraffin oil and Mygliol 812 as the second oil phase. All these materials were medical grade. The oil mixture was stirred at a speed of 427 rpm via a mechanical stirrer (RW16 basic IKA-Werke, Staufen, Germany), a glass rod, and a semicircular stirring blade (*d* = 70 mm). The CaMgP raw powder was thoroughly mixed with 2.0 M (NH_4_)_2_HPO_4_/1.5 M NH_4_H_2_P solution to obtain a homogeneous paste and quickly added into the oil phase under continuous stirring. The stirring process was stopped after 60 min and the granules were left in the oil phase until complete hardening. After removing them from the oil phase, the granules were cleaned by washing them in ultrapure water twice, acetone once, and then again ultrapure water once, for 5 min respectively. Initially, the purified granules were dried at room temperature and then for at least 24 h at 37 °C in an oven. Finally, granules were sieved into different fractions (2000, 1000, 710, 500, 355, and 200 μm) and weighed. Granules of particle size fraction 500 μm–710 μm were used as bone replacement material in the animal study.

As reference material, granules made from a commercially available hydroxyapatite forming bone cement (INNOTERE Paste-CPC, INNOTERE GmbH, Radebeul, Germany) were used. The granules were obtained from fully hardened cement pieces by crushing and sieving to obtain the final particle size fraction of 500–710 μm (CPC granules) instead of using the abovementioned procedure.

### Animal experiments

**Fig. 1:**
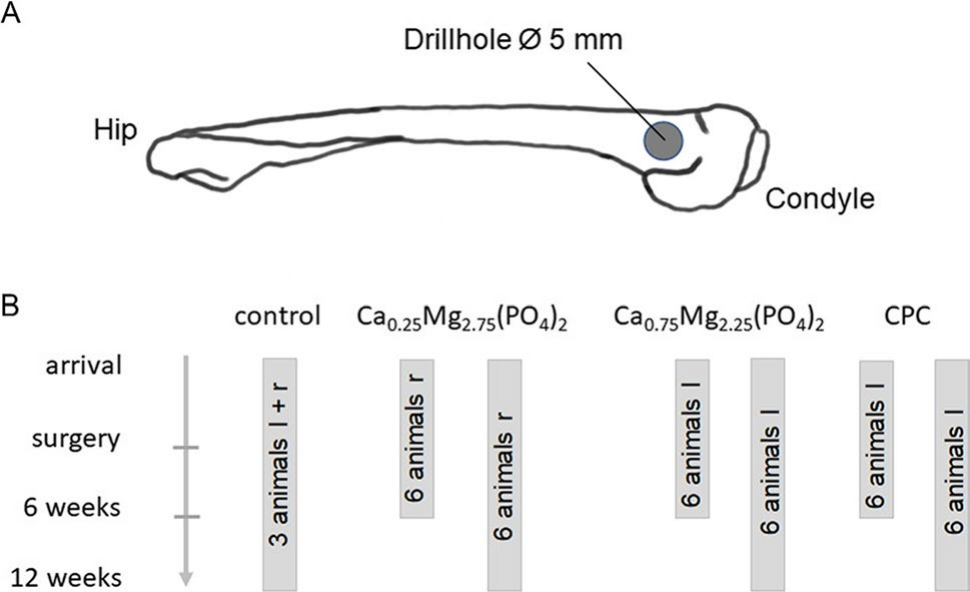
**A** Schematic of the drill hole placement. **B** Overview of the timeline and groups (l: left femur, r: right femur)

The animal experiments were performed under the same conditions as previously reported by our working group [16]. The animal study was approved under § 8 par. 1 of the animal protection act as amended by the act of 04/07/2013 in conjunction with the ordinance on the protection of animals used for experimental purposes of 01/08/2013 after evaluation by an independent ethics commission according to §15 animal welfare act by the local authorities (government of lower franconia, file reference 55.2 2532–2-338) and performed in compliance with international recommendations for care and use of laboratory animals (ARRIVE guidelines and EU Directive 2010/63/EU for animal experiments). The animals were housed in cages (252 cm^2^ × 65 cm) equipped with a raised lying surface and fed with a standard diet (V2333-000, ssniff, Soest, Germany). The room was air-conditioned (19 ± 2 °C). After arrival, the animals were acclimatized for 3 weeks. During this time period, they were accustomed to handling by positive conditioning using fruity bites (Plexx, Elst, Netherlands). Twenty-seven female New Zealand white rabbits (age 13 weeks) with a weight of approx. 3 kg were used and randomly divided into four groups á 6 animals (for each formulation 6 samples per time point). The Mg-containing granules were implanted into one animal at a time, one on each side. The empty defects were set in 3 animals; one on each side. The CPC granules were implanted only in the left femora of animals which were also used as CPC control groups for a different bone replacement material described in a different study [16]. For the implantation of the granules, a drill hole defect was created bilaterally at the distal femoral condyle (Fig. 1A). General anesthesia of the animals was achieved by intramuscular injection of ketamine (60 mg kg^−1^ weight) and xylazine (4 mg kg^−1^ weight), followed by inhalation of isoflurane (CP-Pharma GmbH, Burgdorf, Germany). After shaving and disinfection of the operation site, the skin incision was performed and a bony defect was created at the distal lateral epicondyle (*d* = 5 mm, l = 10 mm). After flushing them with sterile saline, the bone defects were filled with either the granule materials or left empty as a negative control. Finally, the surgical access was sealed by multilayered suture closure. After 6 weeks and 12 weeks post-implantation, the animals were euthanized and the granule implants were explanted (Fig. 1B ).

### Material characterization

Material characterization was performed in a similar manner as reported before [16]. X-ray images of the femora and granule implants were taken via a Bruker Xtreme II imaging system (Bruker Corporation, Billerica, USA). The phase composition of cement raw materials and prepared granules were analyzed by X-ray diffraction (Siemens D5005, Karlsruhe, Germany) using Cu-Kα radiation (40 kV voltage, 40 mA current) in a 2 theta range from 20 to 40°, a step size of 0.02°, and a scan rate of 1.5 s/step. Qualitative evaluation of the diffraction patterns was performed using JCPDS references.

After removing adherent soft tissues and prior to histological analysis, the bone explants were stored in formaldehyde solution for at least 14 days. After embedding the samples in Technovit 7200 (Heraeus Kulzer GmbH, Wehrheim, Germany), slices were cut with a band resaw (Exakt, Advanced Technologies GmbH, Norderstedt, Germany) and grinded down to a thickness of about 20 μm. Then, the slices were stained using the Masson-Goldner-Trichrome staining procedure in order to analyze the contact between implants and surrounding bone tissue (this was done with two slices for each time point and formulation, which means *n* = 12).

To provide quantitative compositional information of the granules and adherent tissues, an energy-dispersive x-ray (EDX) analysis was performed. For this purpose, histology slides were placed into a SEM (Crossbeam, Zeiss, Oberkochen, Germany) equipped with EDX analytics (X-Max ^N^50, Oxford Instruments, Abbington, GB) and the material composition was analyzed using an acceleration voltage of 8 kV.

ImageJ software (NIH, Bethesda, USA) was used to analyze the circumference of the implant materials in contact with mineralized bone, osteoid, or connective tissue. Therefore, 12 slides in total were analyzed per material and time point. The initial granule circumferences were obtained from slices of the starting material embedded in polymethylmethacrylate after grinding as described above. The circumference was determined using a Pen Tablet (Wacom Intuos3 A4, Wacom K.K., Japan) to measure the exact value by retracing the granule in the histological image.

Statistical analysis was performed using SPSS software (IBM SPSS Statistics for Windows, Version 25, Armonk, NY, USA). Statistical differences among the groups were evaluated by applying the independent or the dependent *t*-test depending on whether the implants were in the same animal or not. For all tests, the level of significance was set at *p* < 0.05.

## Results

### General condition of animals

After surgery, all animals showed no limping or obvious signs of pain. Clinical signs of inflammation failed to appear. The intake of food and water was uneventful.

### Size distribution

**Fig. 2:**
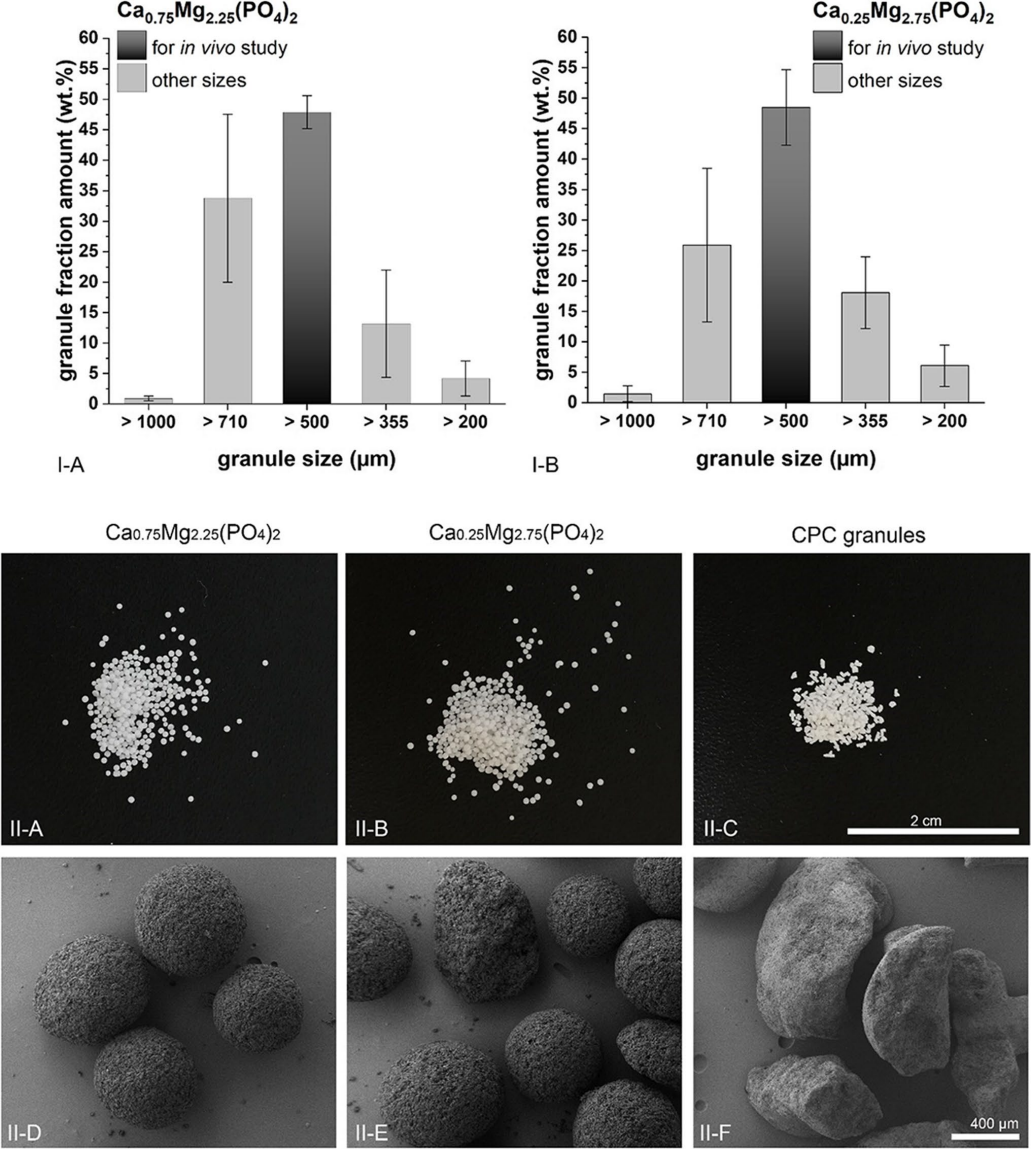
Size distribution of Ca_0.25_Mg_2.75_(PO_4_)_2_ granules (I-A) and Ca_0.75_Mg_2.25_(PO_4_)_2_ granules (I-B) fabricated via the emulsion process. II-A to II-C show pictures of the granule morphology which is shown in more detail in II-D to II-F

After the fabrication of the CaMgP granules, the particles were sieved in order to obtain granule sizes between 500 and 710 μm. Figures 2I-A and I-B show the overall size distribution of CaMgP particles obtained via the described emulsion process. Figures 2II-A–II-C show pictures of the CaMgP granules and the reference CPC granules used in the animal experiments. In Figs. 2II-D–II-F SEM micrographs of the samples are displayed. The CaMgP granules exhibit an ideal spherical morphology without any sharp edges. In contrast, the reference granules are shaped more unsteady.

### X-ray diffraction analysis

**Fig. 3:**
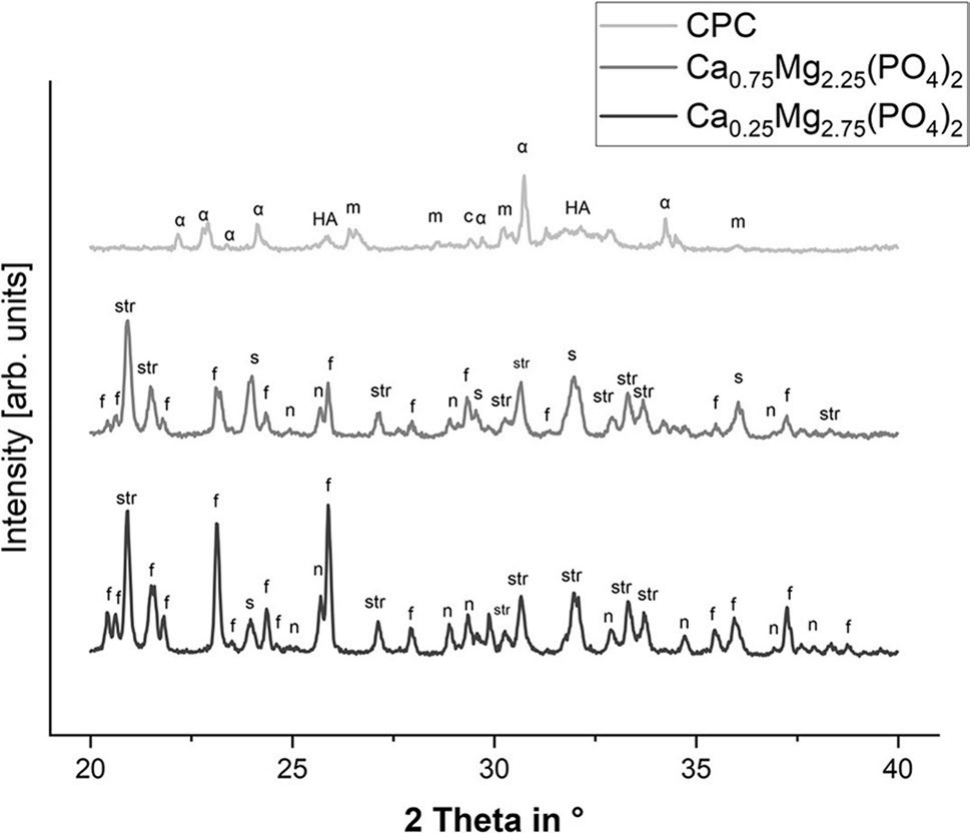
X-ray diffraction patterns of the hardened cement granules; a: β-tricalcium phosphate, HA: hydroxyapatite, f: farringtonite, str: struvite, s: stanfieldite, n: newberyite

X-ray diffraction analysis of the granules (Fig. 3) indicated the presence of nanocrystalline hydroxyapatite and unreacted β-tricalcium phosphate as the main components of the reference material. The composition of the calcium magnesium phosphate granules is somewhere similar to prefabricated cement pastes obtained from the same raw powders [16]. While granules made from Ca_0.25_Mg_2.75_(PO_4_)_2_ were predominantly composed of unreacted farringtonite and the setting products struvite and newberyite, Ca_0.75_Mg_2.25_(PO_4_)_2_ granules additionally contained stanfieldite as residue from the used raw powder.

### Histological findings

**Fig. 4:**
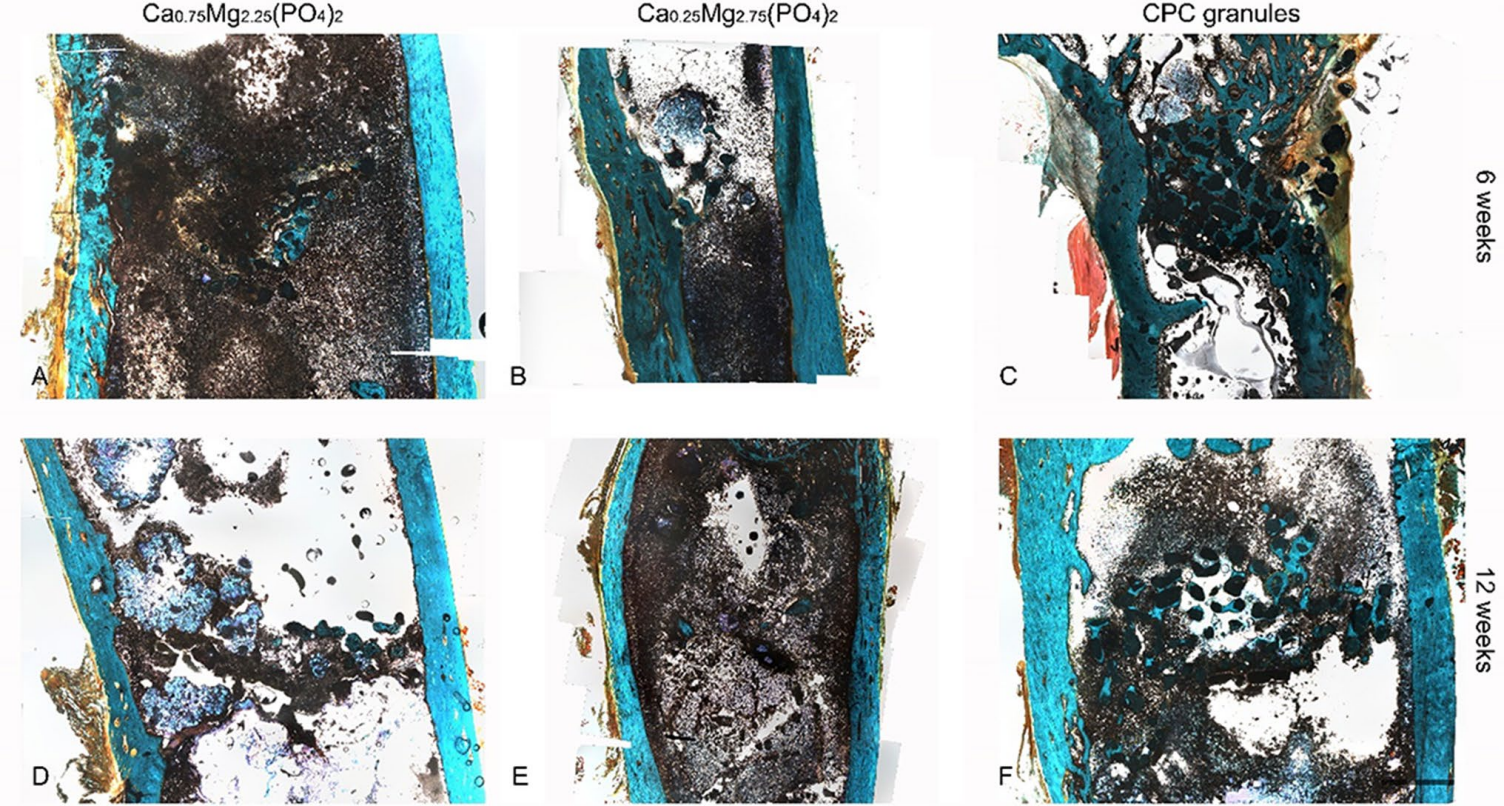
Histological sections of Ca_0.75_Mg_2.25_(PO_4_)_2_ granules (**A**, **D**), Ca_0.25_Mg_2.75_(PO_4_)_2_ granules (**B**, **E**), and CPC granules (**C**, **F**) in the distal femoral epicondyle after 6 and 12 weeks post-implantation. Masson-Goldner-Trichrome staining; *Dark green/black*: granules (some examples are marked with small asterisks), *red*: keratin, muscle tissue, *blue/turquoise*: mineralized bone, *orange*: non-mineralized bone

**Fig. 5:**
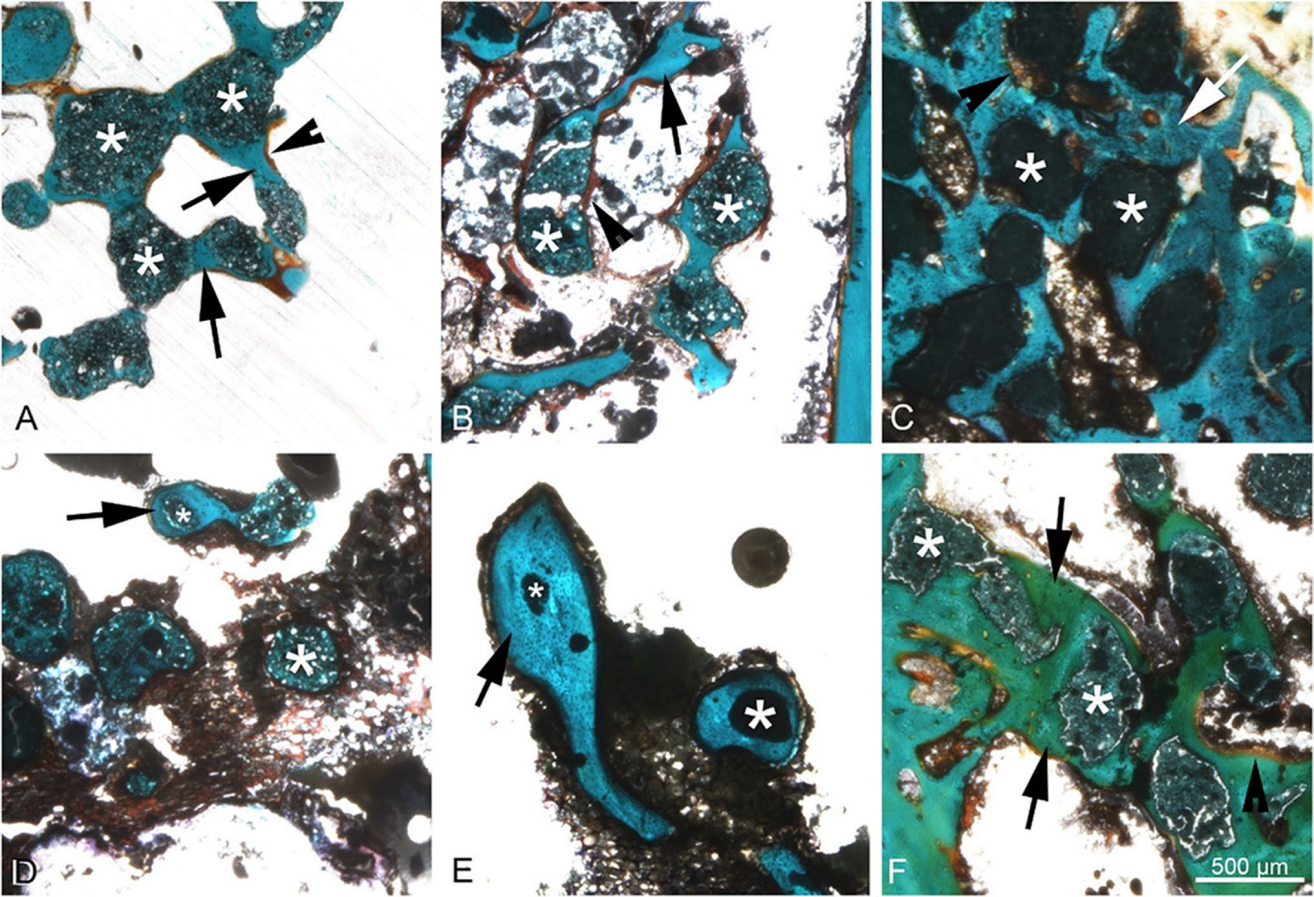
Higher magnification images of the implanted granules. Upper row after 6 weeks, lower row after 12 weeks. Bone replacement material is marked by asterisks. The formed bone around granules is marked by arrows and osteoid by arrowheads. There is no sign of inflammation

Overall, CPC granules (Fig. 4C, F) had a larger diameter than CaMgP granules of both stoichiometries after 6 and 12 weeks of implantation. Besides, the diameter of Ca_0.25_Mg_2.75_(PO_4_)_2_ granules (Fig. 4B, E) did not differ from Ca_0.75_Mg_2.25_(PO_4_)_2_ granules (Fig. 4A, D) significantly at any point. Furthermore, the formation of new bone was observed for all granule types. Especially particles of both CaMgP groups were completely surrounded by newly formed bone tissue in most of the animals. Figure 5 shows the bone-implant contact in higher magnification. The best contact ratio was visible for the control group. But also, both CaMgP groups displayed good bone contact. In some slices of the samples with higher Mg-content, the granules were no longer detectable after 6 weeks of implantation. After 12 weeks of implantation, in both CaMgP variants, granules could no longer be found in most of the slices at all. Generally, for all bone replacement materials used, there were no signs of inflammation. No macrophages or connective tissue capsule could be observed throughout the samples.

### Radiological findings

**Fig. 6:**
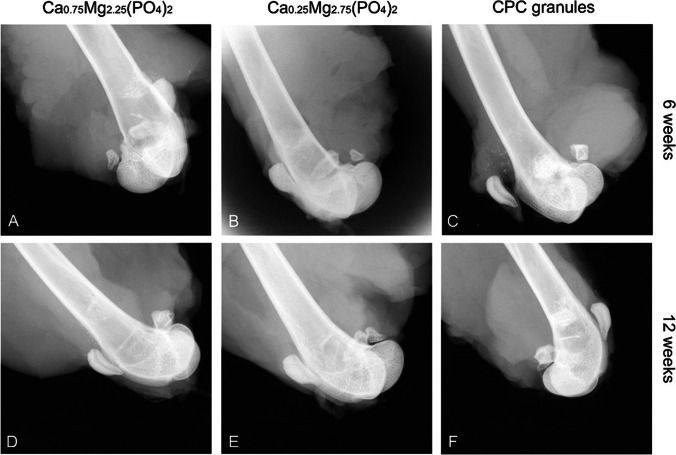
Radiographs of the implanted granules in the distal femoral epicondyle after explantation

X-ray imaging of the implant site revealed no difference in signal strength and granule size for the CPC material after 6 and 12 weeks post-implantation (Fig. 6C, F) with generally high radiopacity in both cases. In contrast, for Ca_0.25_Mg_2.75_(PO_4_)_2_ (Fig. 6B, E) and Ca_0.75_Mg_2.25_(PO_4_)_2_ (Fig. 6A, D), radiopacity decreased considerably from week 6 to week 12. In most radiographs, no more radiological signs of remaining CaMgP materials could be detected at the implantation site after 12 weeks in both material variants tested.

**Fig. 7:**
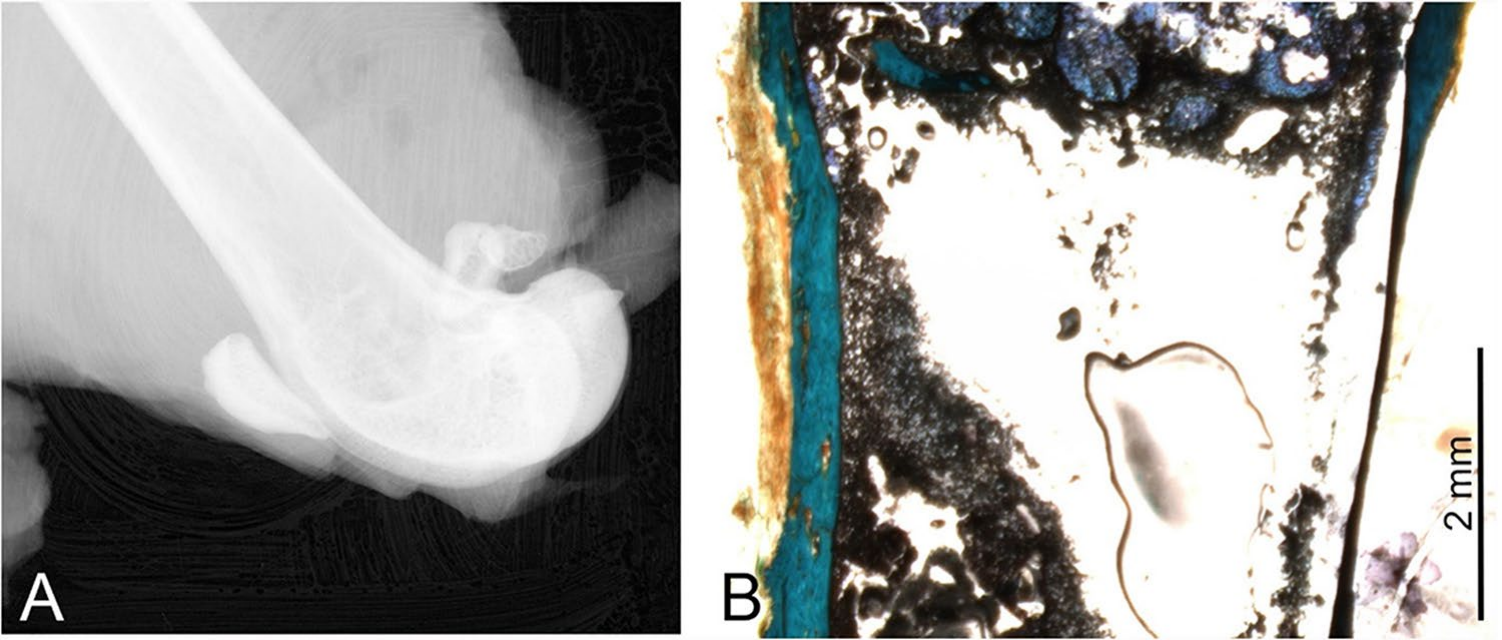
Empty defect after 12 weeks. **A** Radiograph of the defect area. **B** Histology of the defect area. The boundaries of the drill hole can still be detected

For empty defects, after 12 weeks, the defect in the cortical bone was covered by a thin bony lamella. This was indicated by radiological findings (Fig. 7A) and proved by histological findings (Fig. 7B). There was no newly formed bone detectable in the area of bone marrow. In contrast, the newly formed bone could be observed in the bone marrow regions filled with cement granules (Fig. 6).

### Granule degradation

**Fig. 8:**
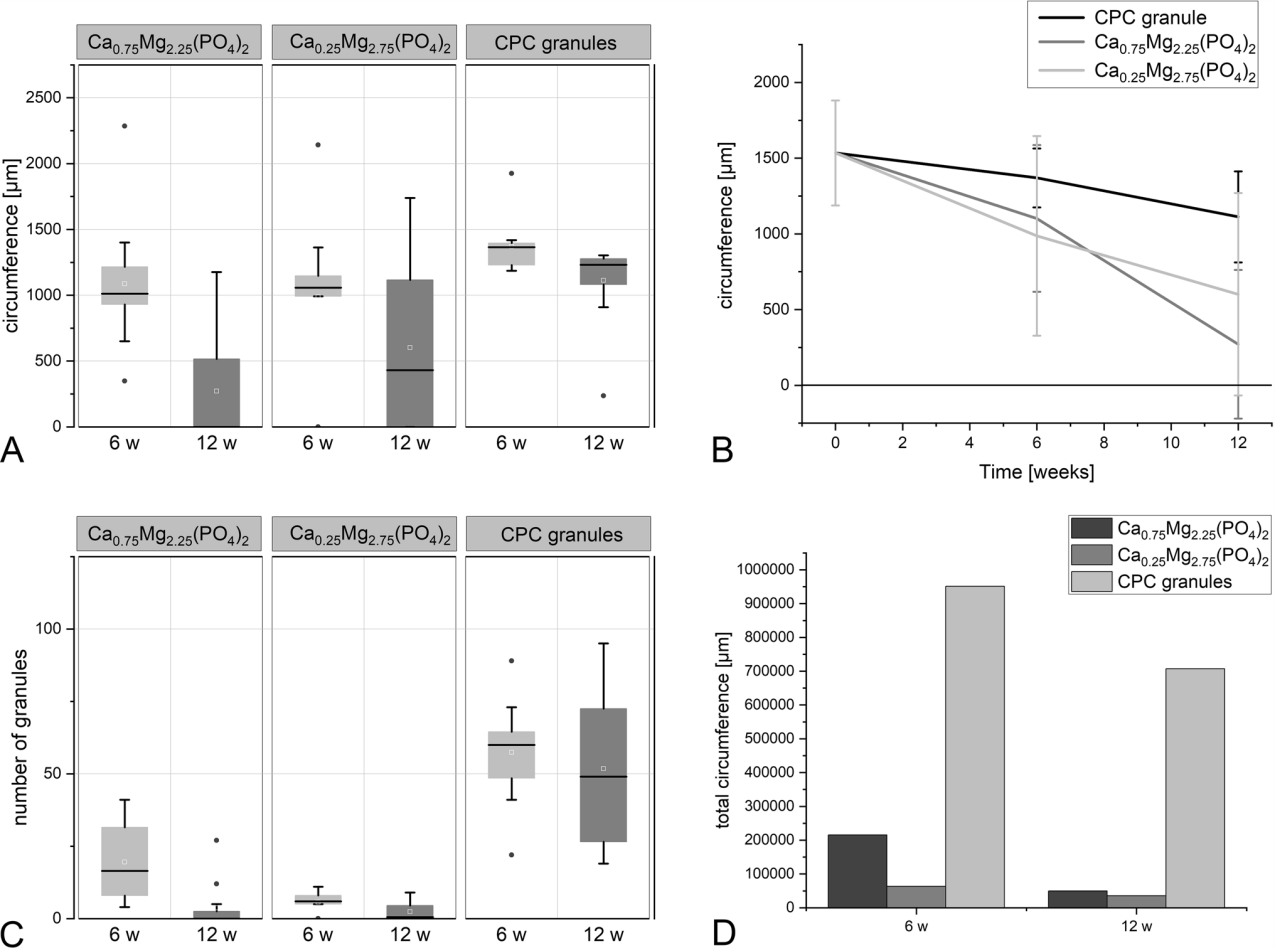
Mean circumference (**A**, **B**), number of granules per slice (**C**), and added circumferences of all granules per sample (**D**) calculated from the histological sections after 6 and 12 weeks of implantation. A total of 12 slices was analyzed per sample

The mean circumference of CPC granules decreased from initially 1500 μm to approx. 1370 μm in 6 weeks and further to approx. 1100 μm after 12 weeks implantation (Fig. 8). For the CaMgP granules, the decrease was more pronounced. Ca_0.25_Mg_2.75_(PO_4_)_2_ granules showed a reduction of circumference from initially 1500 μm to approx. 1000 μm after 6 weeks and further to approx. 600 μm after 12 weeks of implantation. This decrease was even stronger for the Ca_0.75_Mg_2.25_(PO_4_)_2_ granules. Here, the mean particle circumference reduced from initially 1500 μm to approx. 1100 μm after 6 weeks to approx. 270 μm after 12 weeks of implantation. Simultaneously, the number of granules decreased as well, as seen in Fig. 8C. After 12 weeks, granules could hardly be detected in both CaMgP groups. In the Ca_0.25_Mg_2.75_(PO_4_)_2_ samples, granules were found only in 6 slices (28 granules) out of 12, and in Ca_0.75_Mg_2.25_(PO_4_)_2_ samples, only 3 slices (45 granules) out of 12 showed material remnants. Given the added circumference of all granules and the numbers mentioned above, Fig. 8D indicates that there are more small particles leftover in Ca_0.75_Mg_2.25_(PO_4_)_2_ samples than in the Ca_0.25_Mg_2.75_(PO_4_)_2_ samples.

### Osteoid-implant contact

**Fig. 9:**
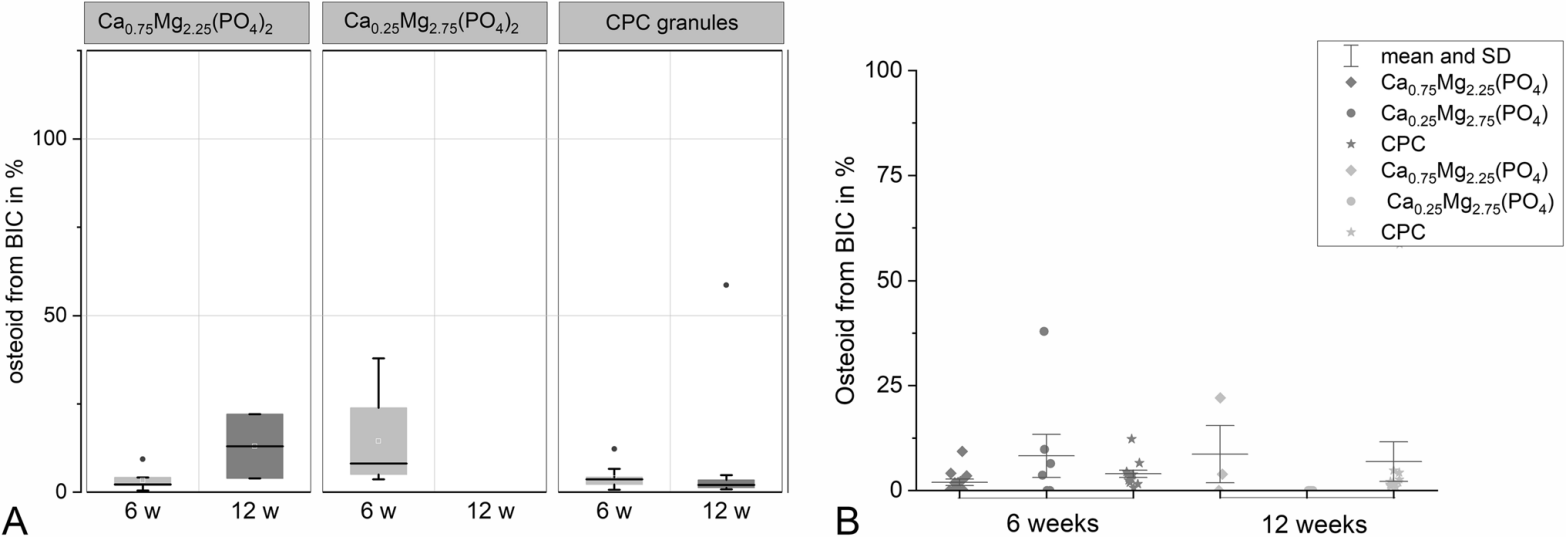
Osteoid-implant contact assessed from the histological sections for CPC and CaMgP granules after 6 and 12 weeks of implantation. There is no significant difference between osteoid proportion after 6 weeks and after 12 weeks

Contact between newly formed osteoid and cement granules did not differ significantly between CPC and CaMgP granules after 6 weeks after implantation. At that time point, the tendentially highest osteoid-implant contact was found for Ca_0.25_Mg_2.75_(PO_4_)_2_ granules (8.2%) followed by CPC granules (3.9%) and Ca_0.75_Mg_2.25_(PO_4_)_2_ granules (1.2%). After 12 weeks of implantation, the osteoid contact in control samples was 6.8% reflecting ongoing mineralization. For Ca_0.75_Mg_2.25_(PO_4_)_2_ samples, only in two slices granules surrounded by osteoid could be found. In contrast, for Ca_0.25_Mg_2.75_(PO_4_)_2_ samples none of the granules was covered with osteoid (Fig. 8C). Consequently, no significant osteoid-implant contact could be observed (Fig. 9A). The distribution of the osteoid percentage in the single slices is shown in Fig. 9B.

### Bone-implant contact

**Fig. 10:**
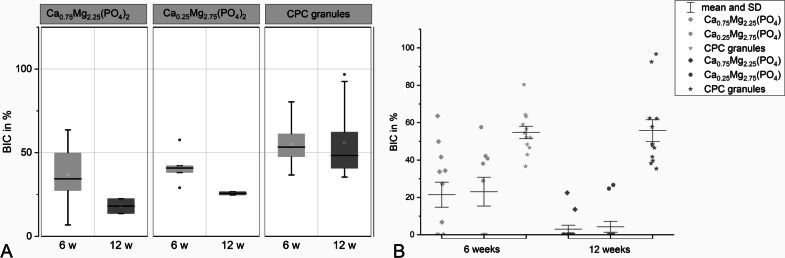
Bone-implant contact assessed from the histological sections for CPC and CaMgP granules after 6 and 12 weeks of implantation. The difference of the CPC granules to Mg-containing samples is significant (*p* < 0.05), whereas there is no significant difference between the Mg-containing samples

Altogether, contact between calcified bone and granule particles was found to be the highest for the CPC control group (54.7% after 6 weeks and 55.8% after 12 weeks of implantation). After 6 weeks of implantation, a good bone-implant contact was observed for the Ca_0.25_Mg_2.75_(PO_4_)_2_ group (23.1%) as well as for Ca_0.75_Mg_2.25_(PO_4_)_2_ group (23.4%) which was significantly lower than the control in both cases (*p* < 0.05). In contrast to the CPC reference, the bone-implant contact decreased slightly for both CaMgP materials until 12 weeks post-implantation. As for osteoid-implant contact evaluation, no significant difference was observed for the two CaMgP groups at this time point as granule particles were mostly no longer detectable (Figs. 5C and 6). The difference to the control granules was significant (*p* < 0.05). Figure 10A displays the values for the bone contact to the granules, which could be detected in some of the sections (in two sections for Ca_0.75_Mg_2.25_(PO_4_)_2_ and six sections for Ca_0.25_Mg_2.75_(PO_4_)_2_). Wherever granule remnants were found, there was bone-implant contact after 12 weeks as well (about 25%). In Fig. 7B, the distribution of the bone.implant contact percentage for every slice is shown in a dot plot.

### EDX measurement

**Fig. 11:**
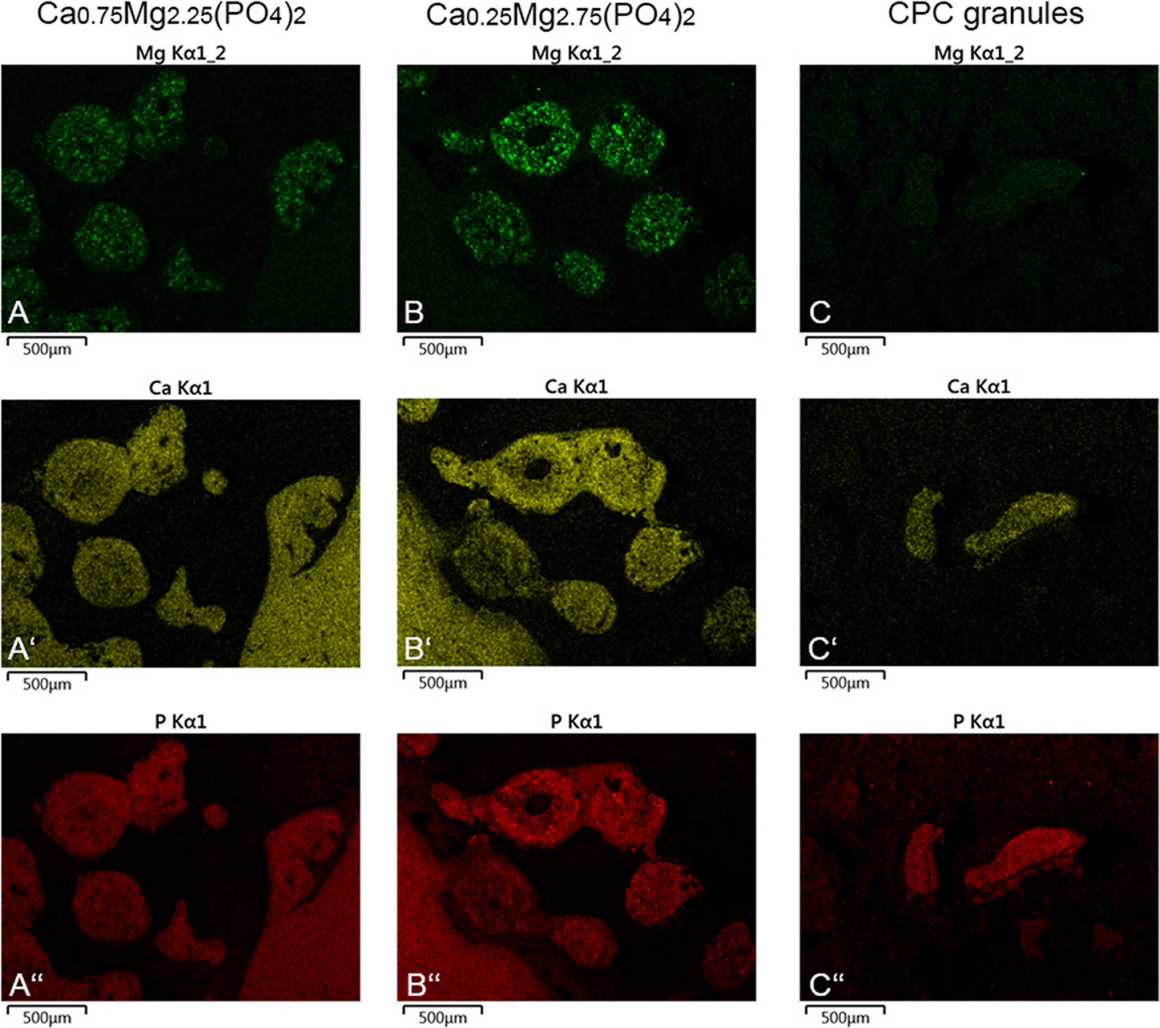
Elemental composition of exemplary implant sites after 6 weeks post-implantation. A–A”: Ca_0.75_Mg_2.25_(PO_4_)_2_- granules, Mg is only detectable in the granules (A), Ca (A’) and P (A”) can be detected in the granules as well as the surrounding bone (lower right). B–B”: Ca_0.25_Mg_2.75_(PO_4_)_2_- granules, Mg is only detectable in the granules (A), Ca (A’) and P (A”) can be detected in the granules as well as the surrounding bone (lower left). C–C”: CPC control granules, only Ca (C’) and P^−^ (C”) was detectable in the granules (and surrounding bone/tissue)

As both, increased material degradation and simultaneous bone formation, were observed for the CaMgP granules during implantation, the Mg content of the newly formed bone was analyzed. As shown in Fig. 11, Mg was only detectable in Mg-containing cement granules (A and B), whereas Ca (A’, B’, C’) and P (A”, B”, C”) were detected in all three granule groups as well as in the surrounding bony tissue.

**Fig. 12:**
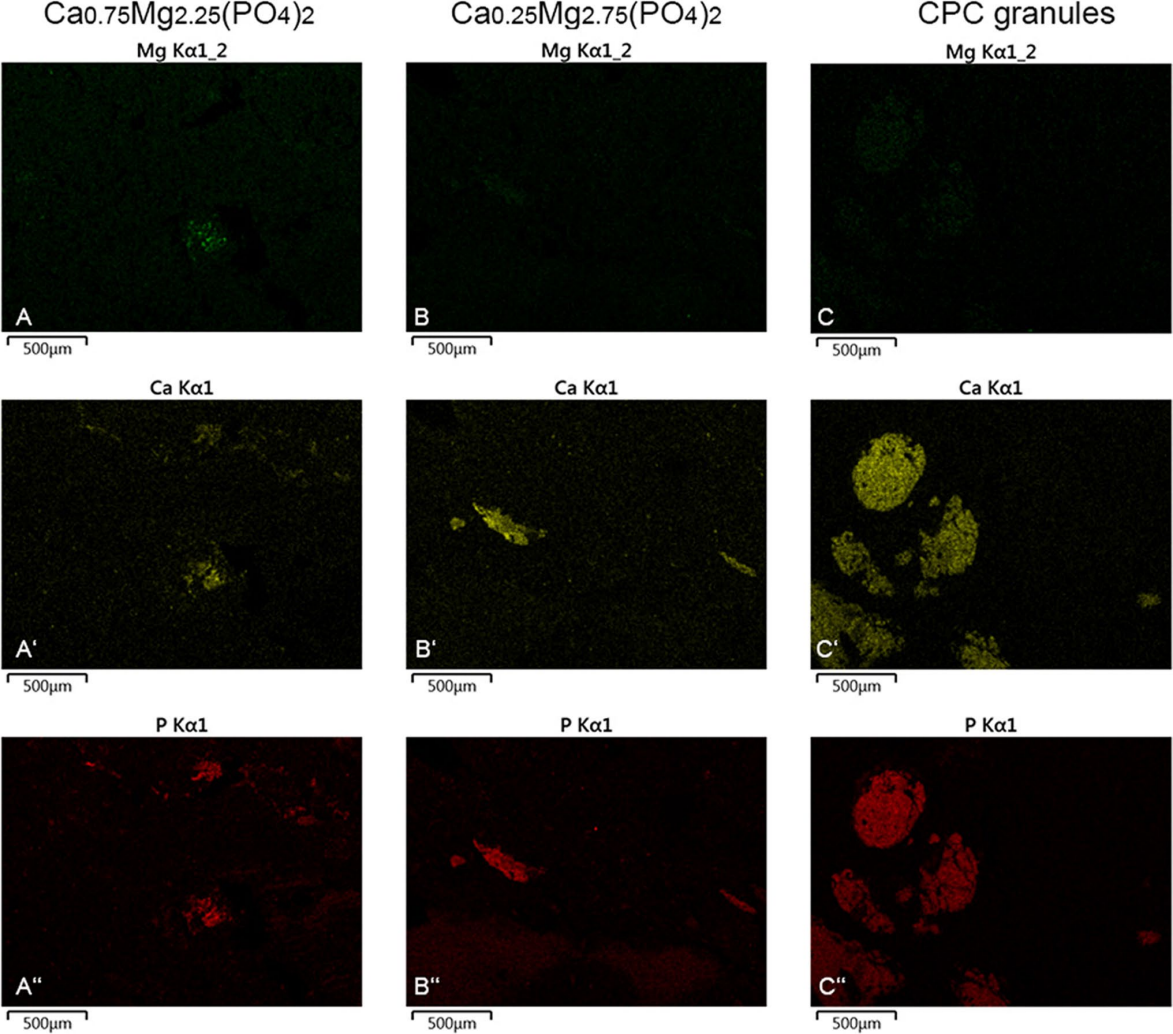
Elemental composition of exemplary implant sites after 12 weeks post-implantation. A–A”: Ca_0.75_Mg_2.25_(PO_4_)_2_- granules, nearly no Mg was detected in the granules (**A**), only weak signals of Ca (A’) and P (A”) were detected. B–B”: Ca_0.25_Mg_2.75_(PO_4_)_2_- granules, no Mg was detected in the granules (**B**), only weak signals of Ca (B’) and P (B”) were detected. C–C”: CPC control granules, still no Mg was detectable (C), Ca (C’) and P (C”) were detectable in the granules as well as in surrounding bone

Twelve weeks after implantation, the element Mg was no longer detectable in the reminiscent CaMgP granules (Fig. 12A, B) and only weak signals of the elements Ca (Fig. 12A’, B’) and P (Fig. 12A”, B”) were observed, showing the advanced degradation of these materials. In contrast, the elements Ca and P were still observed in the CPC control granules (Fig. 12C’, C”) and there were no signs of Mg (Fig. 12C).

## Discussion

Recently, magnesium phosphate cement as well as magnesium-substituted calcium phosphate cement have become more and more interesting as bone substitutes [16, 17]. Compared to the widely used pure calcium phosphate cement, magnesium phosphate cement or magnesium phosphate-doped calcium phosphate cement display several advantages such as high solubility, good biocompatibility, good biomechanical features, and antimicrobial effects, making them ideal material alternatives for bone replacement applications [18, 19].

Bone augmentation via the insertion of particulate bone graft materials is a well-established procedure in oral and maxillofacial surgery, such as sinus floor augmentation of the maxilla [20]. The spectrum of biomaterials used in these indications is vast and includes autografts, allografts, xenografts, and alloplasts partly in combination with growth factors [21]. So far, research on the use of calcium-magnesium phosphate granules for this purpose is limited. Nevertheless, a promising osteogenic potential was already described for magnesium-substituted hydroxyapatite granules in clinical settings [22, 23].

In this study, spherical CaMgP granules with sizes between 500 and 710 μm in diameter were synthesized successfully by the emulsification of cement pastes in a surfactant stabilized lipophilic liquid, as it has been described before for calcium phosphate, as well as for magnesium phosphate–based cement [24, 25]. This way, we fabricated granules of different stoichiometric Mg:Ca ratios which consisted predominantly of unreacted farringtonite and the setting products struvite and newberyite.

In contrast to common fabrication methods like grinding hardened cement monoliths, e.g., as done for the reference CPC granules, the obtained granules displayed no sharp edges [26]. This may be beneficial in maxillofacial applications like the sinus floor elevation, as the perforation of the sinus membrane is one of the most frequently described clinical complication [27]. If perforation of the vulnerable sinus membrane occurs unnoticed or postoperatively, a dislocation of the augmentation material into the maxillary sinus may be the result, which compromises the treatment outcome. In this context, the perforation of the sinus membrane is most frequently caused during the elevation process of the membrane. Perforations of the sinus membrane which can safely be attributed to the augmentation material alone, for example, caused by sharp-edged bone substitutes, have not been reported in the current literature. Nevertheless, the use of atraumatic, spherical granules as a bone substitute as described in this study may further reduce the risk of violating such vulnerable structures. Furthermore, the specific particle size fraction of 500–710 μm can be considered suitable for clinical applications because it is within the size range of one of the most commonly used xenogeneic particulate bone substitute materials (e.g., BioOss, 0.25–1 mm) [28].

The 5-mm drill hole was intended to be a critical size defect according to the current literature [29, 30]. During the experiment, it turned out, that partly, after 12 weeks of implantation, a thin bony lamella was also formed bridging the drill holes of the empty defect group, whereas no signs of newly formed bone could be detected in the bone marrow of the empty defects. In contrast, both CaMgP groups as well as the CPC controls displayed a cortical lamella and signs of bone regeneration within the bone marrow. This suggests at least an accelerated wound healing process within the defect regions.

After their implantation in vivo, radiological investigations revealed a massive loss of radiopacity for Ca_0.25_Mg_2.75_(PO_4_)_2_ as well as for Ca_0.75_Mg_2.25_(PO_4_)_2_–based granulate implants between 6 and 12 weeks. This indicates a strong degradation of the CaMgP–based materials. After 12 weeks of implantation, in some cases, no implant (in 2 out of 12 slices for Ca_0.75_Mg_2.25_(PO_4_)_2_ and in 6 out of 12 slices for Ca_0.25_Mg_2.75_(PO_4_)_2_) but only regularly shaped cortical and trabecular bone was observed in the defect site, and therefore complete bone regeneration can be assumed. This was confirmed for both CaMgP variations by histological findings. All of the magnesium phosphate–based granule implants displayed new bone formation surrounding them. Furthermore, the absence of granules was observed in some of the samples after 12 weeks post-implantation, which indicates a complete degradation of the particles. On the other hand, no significant differences between Ca_0.25_Mg_2.75_(PO_4_)_2_ and Ca_0.75_Mg_2.25_(PO_4_)_2_ groups were observed. In contrast, the calcium phosphate cement control supported proper bone formation as well but showed almost no decrease of radiopacity between the two time points [31]. Therefore, the degradation of CPC-based granules is much slower than magnesium phosphate–based granules. In maxillofacial applications, the degradation time of bone substitutes is an important factor. Ideally, augmentation materials are supposed to be completely replaced by local bone at an absorption rate, which does not lead to a significant loss of tissue volume [3]. An accelerated resorption without the formation of new bone might lead to a failure of the augmentation procedure. For the observed CaMgP granules, we have seen almost complete resorption after 12 weeks of implantation, which is rather quick for bone augmentation materials, where volume constancy is an important requirement. However, at the same time, the newly formed vital bone could be seen within the defect areas, which leads to the assumption, that there is a sufficient volume constancy of implantation sites augmented with CaMgP granules.

Bone-implant contact between calcified bone and bone substitute material was highest in the calcium phosphate cement control groups. This can certainly be attributed to the fact that after 12 weeks post-implantation, only a small number of granules were found for Ca_0.75_Mg_2.25_(PO_4_)_2_ as well as for Ca_0.25_Mg_2.75_(PO_4_)_2_ in some defects, and therefore, no bone-implant contact could be determined. Regarding osteoid-implant contact, no significant differences were observed between all three material groups. Nevertheless, both magnesium phosphate granules showed less osteoid-bone contact than CPC granules, which can also be explained by the increased degradation of the former. The degradation of the bone replacement material seemed very desirable, as it was in a range which allowed new bone formation. This observation was confirmed by the change of granule diameters during the 12 weeks of implantation. While the diameter of CPC granules only decreased by 42%, the diameter of Ca_0.25_Mg_2.75_(PO_4_)_2_ granules decreased by 68% and the diameter of Ca_0.75_Mg_2.25_(PO_4_)_2_ granules by even 85%. In terms of granule volumes, this means that after 12 weeks of implantation, 19% of the calcium phosphate granules remain, while only 3.1% of Ca_0.25_Mg_2.75_(PO_4_)_2_ and 0.33% of Ca_0.75_Mg_2.25_(PO_4_)_2_ are detectable at the implant site.

To date, CaMgP granules have not been examined in vivo considering their potential in bone regeneration, although first in vitro investigations displayed promising material properties [25]. So far, investigations on magnesium-based bone replacement materials focused on 3D printed solid bodies and cement paste formulations [32, 33]. Here, magnesium phosphate–based cement proved that they are able to promote bone regeneration of mechanically stable bone and simultaneous degradation of the cement. Although the herein used MgP implants had different cement formulations and were examined in a large animal model over a longer period of time, histological imaging revealed comparable results with vital newly formed bone embedding the respective cement particles, like we observed for CaMgP granules. At this, a quantitative comparison is hard to make due to the differences in the study designs. Besides and as aforementioned, the examined CaMgP granules differed from the previously examined magnesium phosphate–based cement considering their fabrication. While 3D printed cement solid bodies and, to a lesser extent, formable cement pastes can maintain a certain volume without greater support of the surrounding bone tissue, granules require the presence of multi-walled local bone or at least the use of membranes for bone augmentation purposes. Herein, 3D-printed implants have the particular advantage of an exact preoperative computer-aided planning and design, which neither cement pastes nor granules have. Although a lack of sharp edges of each granule particle might theoretically lead to a decreased mechanical lock of the particles and therefore an increased material dislocation, this could not be observed in practice during the experiments. Overall, clinical handling of the CaMgP granules proved to be very good considering their insertion into the defect areas and was in no way inferior to currently used particulate bone augmentation materials.

As a limitation of the current study, it should be considered that small animal models do not deliver exactly the same information on bone healing as studies in large animals or humans. A generally faster healing due to the faster bone metabolism in rabbits may compromise the findings obtained [34]. Single animals may also display individual and volatile reactions to the respective implants and therefore putting the results into perspective. Nevertheless, especially the rabbit model which was used here, it is a widespread and commonly accepted model to perform screening studies in which new materials can be evaluated considering their materials properties and bone regeneration capacity [35].

In summary, different calcium-magnesium ratios had only little influence on the bone regeneration capacity of the CaMgP granules. Ca_0.25_Mg_2.75_(PO_4_)_2_ as well as Ca_0.75_Mg_2.25_(PO_4_)_2_ formulations displayed very good bone healing properties in vivo. Both granule formulations allowed for sufficient bone ingrowth with a simultaneous fast degradation of the implant material. In this respect, a higher calcium doping seems to cause a slightly faster degradation, whereas the amount of newly formed osteoid and calcified bone seems to be unaffected.

With this study, a first step was made to examine newly developed CaMgP granules with the goal of a prospective use as a bone augmentation material especially for maxillofacial applications. Nevertheless, further investigations have to be conducted. In order to assess bony consolidation of the implants better, three-dimensional imaging modalities like micro-CT of the regions of interest would be desirable. Although resorption of the granules appeared relatively quickly, investigations over a longer period of time may be considered to verify a complete degradation. An implantation into load-bearing defects of larger animals seems not mandatory, as granules are not supposed to be implanted into load-bearing defect situations as they obviously lack initial mechanical stability. In contrast, an integration of CaMgP cement into scaffold structures which are fabricated via 3D printing applications seems a worthwhile option. For direct 3D powder printing of magnesium phosphate cement, various examples already exist, which produce scaffolds with good dimensional accuracy, mechanical features, and cytocompatibility [36, 37]. Another option would be to incorporate small granule particles into polymer scaffold structures which are fabricated using extrusion-based 3D printing systems [32]. Here, a limitation might be the necessary very small granule size in order to pass the printing nozzle and to fit the granules into the oftentimes very fine scaffold structures. In fact, within granule preparation for this study, there have been granule sizes, which were below 200 μm, but they were not examined towards their morphology and they formed only a minority of the obtained granules. Further structural modifications of the cement composition or the emulsification process may increase the fraction of smaller granules.

## Conclusion

By an emulsification of CaMgP cement pastes of different stoichiometric Mg:Ca ratios in a surfactant stabilized lipophilic liquid, completely spherical and therefore atraumatic granules with an ideal size distribution for bone augmentation purposes were successfully synthesized. The resulting calcium-doped magnesium phosphate granules exhibited beneficial material properties as well as excellent clinical handling. The implantation of CaMgP granules in vivo displayed good osseointegration and in some cases a complete resorption of particles and their replacement by vital bone tissue. In contrast, the degradation of granules based on conventional CPC was limited in the investigated defect model. Although the calcium content of the CaMgP formulations had only little influence on its degradation, further modifications may allow for an adjustment of the resorption rate of such materials. In summary, spherical granules based on the presented magnesium phosphate chemistry can be an innovative alternative to commonly used bone augmentation materials made of calcium phosphates, in particular, due to their atraumatic morphology and improved resorbability.

## References

[1] Stumbras A, Kuliesius P, Januzis G, Juodzbalys G (2019). Alveolar ridge preservation after tooth extraction using different bone graft materials and autologous platelet concentrates: a systematic review. J Oral Maxillofac Res.

[2] Dimitriou R, Mataliotakis GI, Calori GM, Giannoudis PV (2012). The role of barrier membranes for guided bone regeneration and restoration of large bone defects: current experimental and clinical evidence. BMC Med.

[3] Sanz M, Dahlin C, Apatzidou D, Artzi Z, Bozic D, Calciolari E, De Bruyn H, Dommisch H, Donos N, Eickholz P, Ellingsen JE, Haugen HJ, Herrera D, Lambert F, Layrolle P, Montero E, al Mustafa K, Omar O, Schliephake H (2019). Biomaterials and regenerative technologies used in bone regeneration in the craniomaxillofacial region: consensus report of group 2 of the 15th European Workshop on Periodontology on Bone Regeneration. J Clin Periodontol.

[4] Jensen SS, Yeo A, Dard M, Hunziker E, Schenk R, Buser D (2007). Evaluation of a novel biphasic calcium phosphate in standardized bone defects: a histologic and histomorphometric study in the mandibles of minipigs. Clin Oral Implants Res.

[5] Anghelescu VM, Neculae I, Dincă O, Vlădan C, Socoliuc C, Cioplea M, Nichita L, Popp C, Zurac S, Bucur A (2018). Inflammatory-driven angiogenesis in bone augmentation with bovine hydroxyapatite, b-tricalcium phosphate, and bioglasses: a comparative study. J Immunol Res.

[6] Iafisco M, Palazzo B, Ito T, Otsuka M, Senna M, Delgado-Lopez JM, Gomez-Morales J, Tampieri A, Prat M, Rimondini L (2012). Preparation of core-shell poly(L-lactic) acid-nanocrystalline apatite hollow microspheres for bone repairing applications. J Mater Sci Mater Med.

[7] Gross KA (2002). Berndt CC (2002) Biomedical application of apatites. Rev Mineral Geochem.

[8] Epple M, Dorozhkin SV (2002). Biological and medical significance of calcium phosphates. Angew Chem Int Ed Engl.

[9] Schmitz JP, Hollinger JO, Milam SB (1999). Reconstruction of bone using calcium phosphate bone cements: a critical review. J Oral Maxillofac Surg.

[10] Mangano C, Piattelli A, Perrotti V, Lezzi G (2008). Dense hydroxyapatite inserted into postextraction sockets: a histologic and histomorphometric 20-year case report. J Periodont.

[11] Ostrowski N, Roy A, Kumta PN (2016). Magnesium phosphate cement systems for hard tissue applications: A review. ACS Biomater Sci Eng.

[12] Tamimi F, Le Nihouannen D, Bassett DC, Ibasco S, Gbureck U, Knowles J, Wright A, Flynn A, Komarova SV, Barralet JE (2011). Biocompatibility of magnesium phosphate minerals and their stability under physiological conditions. Acta Biomater.

[13] Nabiyouni M, Brueckner T, Zhou H, Gbureck U, Bhaduri SB (2018). Magnesium-based bioceramics in orthopedic applications. Acta Biomater.

[14] Wenisch S, Stahl JP, Horas U, Heiss C, Kilian O, Trinkaus K, Hild A, Schnettler R (2003). In vivo mechanisms of hydroxyapatite ceramic degradation by osteoclasts: Fine structural microscopy. J Biomed Mater Res.

[15] Blum C, Brückner T, Ewald A, Ignatius A, Gbureck U (2017). Mg: Ca ratio as regulating factor for osteoclastic in vitro resorption of struvite biocements. Mater Sci Eng C Mater Biol Appl.

[16] Ewald A, Kreczy D, Brückner T, Gbureck U, Bengel M, Hoess A, Nies B, Bator J, Klammert U, Fuchs A (2019). Development and bone regeneration capacity of premixed magnesium phosphate cement pastes. Materials (Basel).

[17] Moseke C, Saratsis V, Gbureck U (2011). Injectability and mechanical properties of magnesium phosphate cements. J Mater Sci Mater Med.

[18] Brueckner T, Heilig P, Jordan MC, Paul MM, Blunk T, Meffert RH, Gbureck U, Hoelscher-Doht S (2019). Biomechanical evaluation of promising different bone substitutes in a clinically relevant test set-up. Materials.

[19] Mestres G, Fernandez-Yague MA, Pastorino D, Montufar EB, Canal C, Manzanares-Cespedes MC, Ginebra MP (2019). In vivo efficiency of antimicrobial inorganic bone grafts in osteomyelitis treatments. Mater Sci Eng C Mater Biol Appl.

[20] Starch-Jensen T, Jensen JD (2017). Maxillary sinus floor augmentation: a review of selected treatment modalities. Oral Maxillofac Res.

[21] Danesh-Sani SA, Loomer PM, Wallace SS (2016). A comprehensive clinical review of maxillary sinus floor elevation: anatomy, techniques, biomaterials and complications. Br J Oral Maxillofac Surg.

[22] Crespi R, Mariani E, Benasciutti E, Capparè P, Cenci S, Gherlone E (2009). Magnesium-enriched hydroxyapatite versus autologous bone in maxillary sinus grafting: combining histomorphometry with osteoblast gene expression profiles ex vivo. J Periodontol.

[23] Crespi R, Capparè P, Gherlone E (2010). Osteotome sinus floor elevation and simultaneous implant placement in grafted biomaterial sockets: 3 years of follow-up. J Periodontol.

[24] Kim YH, Son SR, Sarkar SK, Lee BT (2014). The effects of dimethyl 3,3'-dithiobispropionimidate di-hydrochloride cross-linking of collagen and gelatin coating on porous spherical biphasic calcium phosphate granules. J Biomater Appl.

[25] Christel T, Geffers M, Klammert U, Nies B, Höß A, Groll J, Kübler AC, Gbureck U (2014). Fabrication and cytocompatibility of spherical magnesium ammonium phosphate granules. Mater Sci Eng C Mater Biol Appl.

[26] Tamimi FM, Torres J, Tresguerres I, Clemente C, López-Cabarcos E, Blanco LJ (2006). Bone augmentation in rabbit calvariae: comparative study between Bio-Oss and a novel beta-TCP/DCPD granulate. J Clin Periodontol.

[27] Stacchi C, Andolsek F, Berton F, Perinetti G, Navarra CO, Di Lenarda R (2017). Intraoperative complications during sinus floor elevation with lateral approach: a systematic review. Int J Oral Maxillofac Implants.

[28] Fujisawa K, Akita K, Fukuda N, Kamada K, Kudoh T, Ohe G, Mano T, Tsuru K, Ishikawa K, Miyamoto Y (2018). Compositional and histological comparison of carbonate apatite fabricated by dissolution-precipitation reaction and Bio-Oss®. J Mater Sci Mater Med.

[29] Levingstone TJ, Thompson E, Matsiko A, Schepens A, Gleeson JP, O’Brien FJ (2016). Multi-layered collagen-based scaffolds for osteochondral defect repair in rabbits. Acta Biomater.

[30] Sarahrudi K, Mousavi M, Grossschmidt K, Sela N, König F, Vécsei V, Aharinejad S (2008). Combination of anorganic bovine-derived hydroxyapatite with binding peptide does not enhance bone healing in a critical-size defect in a rabbit model. J Orthop Res.

[31] Ooms EM, Wolke JGC, van de Heuvel MT, Jeschke B, Jansen JA (2003). Histological evaluation of the bone response to calcium phosphate cement implanted in cortical bone. Biomaterials.

[32] Golafshan N, Vorndran E, Zaharievski S, Brommer H, Kadumudi FB, Dolatshahi-Pirouz A, Gbureck U, van Weeren R, Castilho M, Malda J (2020). Tough magnesium phosphate-based 3D-printed implants induce bone regeneration in an equine defect model. Biomaterials.

[33] Kanter B, Vikman A, Brückner T, Schamel M, Gbureck U, Ignatius A (2018). Bone regeneration capacity of magnesium phosphate cements in a large animal model. Acta Biomater.

[34] Wancket LM (2015). Animal models for evaluation of bone implants and devices: Comparative bone structure and common model uses. Vet Pathol.

[35] Stuebinger S, Dard M (2013). The rabbit as experimental model for research in implant dentistry and related tissue regeneration. J Investig Surg.

[36] Klammert U, Vorndran E, Reuther T, Müller FA, Zorn K, Gbureck U (2010). Low temperature fabrication of magnesium phosphate cement scaffolds by 3D powder printing. J Mater Sci Mater Med.

[37] Meininger S, Moseke C, Spatz K, März E, Blum C, Ewald A, Vorndran E (2019). Effect of strontium substitution on the material properties and osteogenic potential of 3D powder printed magnesium phosphate scaffolds. Mater Sci Eng C Mater Biol Appl.

